# Trading Imbalance in Chinese Stock Market—A High-Frequency View

**DOI:** 10.3390/e22080897

**Published:** 2020-08-15

**Authors:** Shan Lu, Jichang Zhao, Huiwen Wang

**Affiliations:** 1 School of Statistics and Mathematics, Central University of Finance and Economics, Beijing 100081, China; shan.lu@cufe.edu.cn; 2 School of Economics and Management, Beihang University, Beijing 100191, China; hwwang@buaa.edu.cn; 3 Beijing Advanced Innovation Center for Big Data and Brain Computing, Beijing 100191, China

**Keywords:** stock market crash, trading behavior, trading imbalance, trading polarity

## Abstract

Although an imbalance of buying and selling profoundly affects the formation of market trends, a fine-granularity investigation of this perplexity of trading behavior is still missing. Instead of using existing entropy measures, this paper proposed a new indicator based on transaction dataset that enables us to inspect both the direction and the magnitude of this imbalance at high frequency, which we call “polarity”. The polarity aims to measure the unevenness of the very essence trading desire based on the most micro decision making units. We investigate the relationship between the polarity and the return at both market-level and stock-level and find that the autocorrelated polarities cause a positive relation between lagged polarities and returns, while the current polarity is the opposite. It is also revealed that these associations shift according to the market conditions. In fact, when aggregating the one-minute polarities into daily signals, we find not only significant correlations disclosed by the market polarity and market emotion, but also the reliability of these signals in terms of reflecting the transitions of market-level behavior. These results imply that our presented polarity can reflect the market sentiment and condition in real time. Indeed, the trading polarity provides a new indicator from a high-frequency perspective to understand and foresee the market’s behavior in a data-driven manner.

## 1. Introduction

In the 2015 Chinese stock market crash, one-third of the A-shares market value was evaporated abruptly within one month after huge volumes of panic sell-offs. This event underlines the non-negligible roles of the trading behavior, especially when inexperienced investors dominate the market [1]. A stock market crash is usually triggered by economic events, but it is subsequently led by crowd behavior and psychology such as mimicking trading fashions [2,3,4] and severe overlap of portfolios [5,6]. Among these trading behaviors, one of the most important aspects behind the market microstructure is the imbalance of selling and buying parties.

An intuitive measurement of imbalance, called herding, was first proposed to study the trading behavior of institutions [7]. Herding accounts for the inequality between the number of managers who cut their holdings and the number of those who increase their holdings at the level of individual stocks with quarterly frequency. This measurement has been widely used in subsequent studies [8,9]. When examining individual investors’ trading patterns, a similar measurement is also applied to measure the marginal difference in the extent to which an investor’s sales of a stock tend to parrot other individual investors’ tendencies to sell the stock in the subsequent trading days [10]. A related concept, known as the individual investor imbalance, quantifies the net individual trading effect, and it has been proved to have predictive power with respect to abnormal returns on and after earning announcements [11,12]. The existing findings suggest that the imbalance of selling and buying can be indicative or even predictive in understanding market trends.

Nevertheless, the above two branches of measuring imbalance need the identities of investors, which are rarely available in common stock transaction datasets for the protection of privacy. Moreover, these measures only investigate one type of investor, namely, either institutional or individual investors. To address whether the seller initiates a trade or not, a measurement called the order imbalance has been defined to evaluate the inequality of the market control power by either the selling party or the buying party [13]. Interestingly, these authors have demonstrated that when the buyer-initiated orders overwhelm the seller-initiated orders on one trading day, the returns will be higher on the next trading day. Thus, instead of exploring the imbalance at the investor level, drilling down by probing the imbalance at the granularity of order could be a new proxy measurement to examine the overall stocks as well as the market with rich details reserved. The imbalance at the granularity of order delivers information on the perplexity of trading behavior and unevenness of trading desire, which can be measured by entropy, for example, Shannon entropy. However, the entropy only preserves the magnitude of imbalance without the direction that indicates the trading demand surpass supply or the other way round. Yet, the direction of imbalance is genuinely of great importance because it unveils the essential tendency of traders’ expectations.

Motivated by the above, we derive an indicator called trading polarity by taking advantage of the transaction-level data in the Shenzhen Stock Exchange. The trading polarity indicator is defined as the difference between the buying and selling man-times rescaled by the total man-times involved in a given time interval. Unlike the imbalance indicators defined in the previous studies, which usually only reflect the imbalance of one type of investor and at best on a daily basis, the presented indicator particularly reflects the degree of imbalance in selling and buying from a man-times viewpoint, particularly at high frequency. Similar to other entropy measures, the proposed indicator works for capturing the micro-state of system dynamics, in particular, the stocks’ trading sequences, at high frequency. It is worth noting that the man-time of a transaction is the most micro unit to depict trading behavior, and it captures the minimum decision unit of market participants without leaking the traders’ identity. Moreover, as we measure the trading polarity on a one-minute basis, the indicator provides not only cross-sectional but also longitudinal detailed information on the imbalance phenomenon; this information is quite useful under the current circumstance of a wide range of high-frequency trading and financial big data [14,15,16]. As a result, the defined indicator could offer new insights into the behavior and characteristics of a market.

As the trading polarity reflects excessive investor interest in stocks, it could be related to future returns and provide additional power beyond trading activity measures for explaining the stock market. Although many studies have investigated the imbalance outside of crisis periods [10,11,12,13], we will specifically explore the strength and influence of the imbalance between selling and buying during a stock market crash. We start with the summary statistics of the trading polarity and find that the one-minute polarities are positively autocorrelated both at market-level and stock-level. Investigations on the relationships between the polarity and the return at the two levels show that the autocorrelated polarities cause a positive relation between lagged polarities and returns, while the current polarity has the opposite effect. These results are consistent with previous studies and imply that the theory of inventory effects in stock price movements still hold at the high-frequency facet. More importantly, though the polarity could only captures the imbalance in the corresponding small time window as it is refined from transactions during one minute interval, we find that the fine-grained polarities impact prices for 3 min, which cannot be ignored, especially for high-frequency trading. Additionally, it is shown that this correlation changes daily according to the market conditions at the stock level, suggesting the polarity could work as a reliable signal that originates from trading decisions at the micro-level for the dynamics of aggregate system at the macro-level.

Given the above, we further explore how to extract useful signals from polarity to capture the market transitions. We use concepts from econophysics, as studies in econophysics in recent decades have shown their advantages for understanding the global behavior of economic systems without the preparation of a detailed microscopic description of the same system [17,18,19,20,21]. We utilize statistical concepts, such as the power-law distribution and burstiness, to investigate the stock market system at different scales. We find that the trading polarity successfully illustrates the transitions of a market’s condition in terms of its flipping depth. In addition, the length before the polarity flipping follows a roughly stable power-law distribution that exhibits the underlying rule behind trading activity. Moreover, the observed bursty character of length before a polarity flip reflects the potentially generic feature of trading dynamics. Even more inspiring, the significant correlation disclosed by the market polarity and market emotion implies that our presented polarity, which is essentially calculated in the context of high-frequency trading data, can reflect the sentiment of the market in real time. Therefore, we argue that the trading polarity provides a new way to understand and foresee the market’s behavior.

## 2. Methods and Materials

### 2.1. Trading Polarity

**Figure 1:**
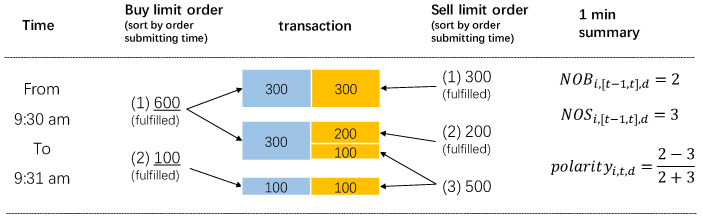
Illustrations of how polarity is calculated. For simplicity purpose, suppose that the presented limit orders are all submitted from 9:30 am to 9:31 am (within one min interval) for stock *i* on day *d*. All the limit orders are at the same price and are ordered according to the quote sequence. We view one order as one man-time, which is the most micro decision unit in stock market. There are two buy limit orders and three sell limit orders involved in the three transactions happened, indicating $NOB_{i,[t-1,t],d}=2$ and $NOS_{i,[t-1,t],d}=3$. Note that the third sell limit order is not fully fulfilled and might be involved in the next minute.

When the transactions are partially filled, there will be an imbalance. For a given stock *i* in time interval [t−1,t] on day *d*, we define the trading polarity as

(1) $${\mathrm{polarity}}_{i,t,d}=\frac{NOB_{i,[t-1,t],d}-NOS_{i,[t-1,t],d}}{NOB_{i,[t-1,t],d}+NOS_{i,[t-1,t],d}},$$

where NOB denotes the number of buying man-times, NOS denotes the number of selling man-times. This indicator can reflect the imbalance of selling and buying man-times in a specific time interval [t−1,t] during a trading day. Because the polarity is scaled by the total number of buying and selling man-times of stock *i* in [t−1,t], it is possible to compare the extent of the imbalance among different time intervals and among different trading days regardless of the market environment. The polarity is also comparable among stocks, as the indicator ranges from −1 to 1. A positive polarity reveals that the buying man-times overwhelm the selling man-times. More specifically, a positive polarity denotes an imbalance between buyers and sellers in which the buying man-times exceed the selling man-times, what we call “buying polarity”. In this situation, there is a net buying man-times flow. A negative polarity, what we call “selling polarity”, is the imbalance in which the selling man-times exceed the buying man-times. And in accordance with this, there is a net selling man-times flow. Considering the fact that in a realistic dataset, only transactions that had already been conducted are recorded, the imbalance in the number of trading parties could reflect the following: (1) given a certain trading volume, the sign of the polarity reveals whether the trading is concentrated in the hands of fewer sellers than buyers or vice versa; (2) the concentration of the transactions, or the extent of the imbalance, is revealed by the absolute value of the polarity. Figure 1 shows an example. As can be seen, the volumes of orders are already embedded in the definition of polarity as the same volume of selling orders and buying orders are meant to form transactions.

Broadly speaking, the trading polarity indicates which party is more crowded or potentially has more investors that agree on whether it is time to sell or to buy. For instance, the indicator can be high either due to a wide range of investors who are buying the stock or a small number of investors who are selling it. Thus, the polarity could measure how market participants anticipate the price trend as well as the outcome of multi-player gaming with trading under complex circumstances within the specific time interval. In other words, the net number of trading man-times (which includes all market participants, such as institutional and individual investors) indicates the market sentiment toward stocks.

Since in this study it is anticipated to establish a new measure of high frequency that could inherently reflect the perplexity of the trading in the stock market, we can fulfill it by utilizing entropy measures to represent the unevenness of buying and selling in trading decisions that revealed in transaction data. Following the definition of Shannon Entropy, here we define the entropy of the man-times trading imbalance as

(2) $${\mathrm{entropy}}_{i,t,d}=-P_{buy}\log\left(P_{buy}\right)-P_{sell}\log\left(P_{sell}\right),$$

where $P_{buy}=NOB_{i,[t-1,t],d}/\left(NOB_{i,[t-1,t],d}+NOS_{i,[t-1,t],d}\right)$, $P_{sell}=NOS_{i,[t-1,t],d}/\left(NOB_{i,[t-1,t],d}+NOS_{i,[t-1,t],d}\right)$. For example, the entropy would be $entropy=-\frac{2}{5}\log\left(\frac{2}{5}\right)-\frac{3}{5}\log\left(\frac{3}{5}\right)$ in Figure 1. As can be seen, the proposed polarity, by definition, is an analogy of entropy. They both take the probability of selling and buying into consideration to characterize the unevenness of microstates for demand and supply in stock trading, and thus work as an expression of the perplexity of the trading in the stock market.

**Figure 2:**
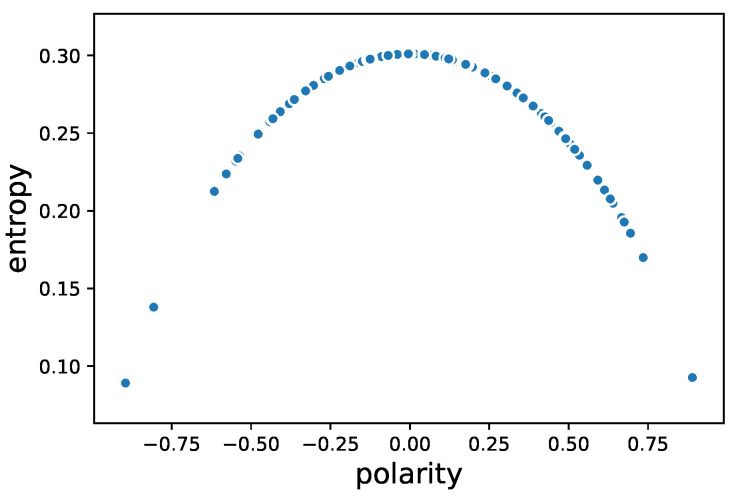
The relationship between ${\mathrm{entropy}}_{i,t,d}$ and ${\mathrm{polarity}}_{i,t,d}$. For the convenience of visualization, here we take the stock “000001.SZ” on 8 May 2015 for example. The two indicators are on a one-minute basis.

Figure 2 shows the relationship between ${\mathrm{entropy}}_{i,t,d}$ and ${\mathrm{polarity}}_{i,t,d}$ where we take the stock “000001.SZ” on 8 May 2015 for example. Note that the entropy is always greater than zero, while the polarity ranges from − 1 to 1 with the sign indicating direction of imbalance. We deem it crucial for an imbalance measure to acquire imbalance direction into quantifying the microscopic characteristic of trading, rather than treating the different imbalance directions as the same. In fact, the change of polarity signs, what we called “flipping” in this study, could reflect the micro-oscillations to a bull or bear market, as we will show in Section 3.4. Therefore, we adopt the polarity in the rest of analysis, which depicts the state a trading dynamic system in a specific time interval, with absolute value referring to the perplexity of the trading decisions and signs referring to which side dominating the trading.

While trading imbalance measures based on volumes or orders are very informative in picturing investor trading behavior and have been used in previous studies, for example, Lakonishok et al. [7], Wermers [8], Sias [9], Grinblatt et al. [10], Kaniel et al. [11,12], Chordia and Subrahmanyam [13], Zhang et al. [22], the tick data that used in these studies either include identities of investors (investors’ ID, individual or institutional investors) or trading direction (buyer-initiated or seller-initiated), which are unfortunately not available in our dataset. In contrast to those definitions that regard sellers or buyers as different types of trader, we argue that the number of buying man-times and the number of selling man-times represent the very essence of the trading desire in an extremely short time. That is, we treat the man-times as the most micro decision unit of the transaction regardless of whether they are conducted by one or more investors, which is crucial to explaining the imbalance of the trading parties. In particular, considering there’s no need of trader information that might infringe the privacy, our newly presented measure could more flexible and feasible than previous indicators. In addition, our methods and analysis framework can be readily adapted to any other markets where the transactions data with order sequential number are available.

### 2.2. Data

#### 2.2.1. Sample Period: 2015 Stock Market Crash of China

**Figure 3:**
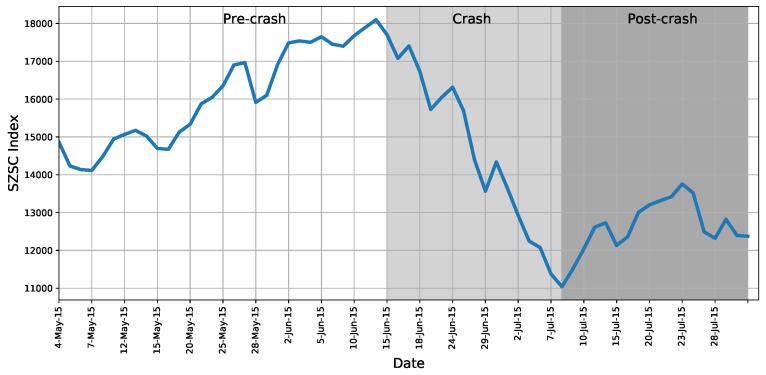
The Shenzhen Stock Exchange Component (SZSC) index from 4 May 2015 to 31 July 2015. The SZSC index is an index of 500 stocks that are traded at the Shenzhen Stock Exchange. The index shows that the market experienced a large rise and fall in this segment. On 12 June, there was a peak at 18098.27 points. From 15 June to 6 July, this figure experienced a sharp decline. Thereafter, the index reached its lowest value in July and rose slightly from 8 July to 31 July. We accordingly cut the sample period into three parts in this order, and they are indicated by different backgrounds.

To sufficiently illustrate the power of trading polarity, targeting a period with market performing ups and downs to sample transactions would be ideal. It has been shown that the systemic risk was higher during the crashes in 2001 and 2008 than in calm periods for the China stock market [23]. The 2015 China stock market crash is generally considered the worst stock market crash since 2007. As we aim to focus on the behavior around the market crash, we narrow down the sample period to 4 May 2015 till 31 July 2015. As can be seen in Figure 3, the stock market experienced a large rise and fall in this interval, as revealed by the Shenzhen Stock Exchange Composite (SZSC) index. Initially, people trusted the market, and this trust coincided with a subsequent rise in prices. It is shown by the SZSC index that May 5 to June 12 witnessed an unprecedented bull market as the index continued rising up to the ending of what we call the “pre-crash” period.

As the stocks became over-priced, the bull market came to an end, and the beginning of the crash occurred on 15 June 2015. The market participants went from not believing the market was declining to an abrupt panic sell-off that suddenly triggered sharply falling prices; another panic followed. Roughly speaking, the first throes of the crash ended around 7 July 2015. We call this period the “crash” period.

The second throes of the crash occurred from 8 July to 31 July. In this period, the government had enacted many measures such as limiting short selling and making mutual funds pledge to buy more stocks [24]. However, those tremendous measures were of little success, revealed by only a small rise in the stock index, as shown in Figure 3. We call this period the “post-crash” period.

The three-month period contains both a bull market and a bear market, which is ideal for the present study. In the following analysis, the sample is divided into three parts in the chronological order described above.

#### 2.2.2. Transaction Records

The data employed in the study contain those transactions that occurred on the Shenzhen Stock Exchange between 4 May 2015 and 31 July 2015, and they cover 1,471,848,085 records of transactions and 1646 stocks. The dataset consists of the stock ID, price, number of shares traded, time for every trade, and, most importantly, the serial numbers for selling and buying orders. Unlike the previous studies, the uniqueness of our data is such that for each selling or buying order (a one-time order involves the price and volume for one stock), an identical serial number will be assigned to it according to the quote sequence. When the quote order is fully or partially fulfilled, the serial numbers of the buying and selling orders are recorded in the transaction record. If the order is partially fulfilled, the serial number could appear multiple times in the transaction records, as it is fulfilled by several counterparties’ orders, as shown in Figure 1. Consequently, the serial numbers are reset at the beginning of the trading day, and they depend solely on the order time regardless of the stocks. The serial numbers enable us to distinguish potentially different trading decision units and count the number of man-times for buying and selling if we are given the transaction records in a specific time interval. Otherwise, one may argue that “for every buyer, there is a seller”.

Admittedly, this paper is not the first to employ transactional data in a financial study. Most of the datasets in these studies consist of stock IDs, trading prices and volumes, and the timestamps of trading [15,25,26,27,28]. However, to the best of our knowledge, this paper is the first to employ man-times selling and buying data. There are at least two advantages to using man-times information to capture the imbalance between buying and selling. First, the serial numbers of buyers and sellers could be available in transactional data not only in China but also in other countries without leaking the privacy of traders. To our knowledge, there are a limited number of studies in the stock market field that contain the traders’ information in their dataset [10,29,30]. In fact, not all countries (including China) allow the data with traders’ IDs to go public even within a small range of financial companies. Using man-times makes it easier for the imbalance indicator to be applied in practice, especially in the big data era. Second, using man-times provides a more systematic view to measure the imbalance of the whole market. In other words, instead of emphasizing trader-level individual effects on transaction activity, the man-times level of trading polarity treats every trading decision unit equally and integrates them together to offer a global perspective on trading activities. Although the trading polarity originates from “micro” data, it could reflect the “macro” landscape of the stock market imbalance. In the results section, we show the usefulness of the proposed indicator in explaining the whole market picture.

To be more specific, we compute the polarity for each stock at a one minute frequency. For simplicity, only the consecutive trading hours are included in the sample; they range from 9:30 am to 11:30 am and from 13:00 pm to 14:57 pm, which amounts to 237 min per day. Thus, we have the polarity time series for 1646 stocks listed on the Shenzhen Stock Exchange for analysis (the sample has excluded stocks that had no trading at all during the sample period). We argue and show in the rest of the paper that the proposed indicator is an effective and practical tool with which to observe the market microstructure in high-frequency data and profile the market macrodynamic by bottom-up aggregation.

#### 2.2.3. Stock Prices

The data on the stock prices were downloaded from Thomson Reuters’ Tick History. Two types of price time series data are used—*end-of-day* and *intraday*. We use the closing price from the *end-of-day* data, as it is the baseline price for computing the daily return for the next day, which is denoted as $p_{i,d}$. The daily percentage change of stock *i* on day *d* is computed by $\left(p_{i,d}-p_{i,d-1}\right)/p_{i,d-1}$. The reason is that this type of percentage change is consistent with what investors see during trading days on any trading information board, which could stir up tensions and impact the prices of stocks directly through trading behavior. Moreover, we use the last price of every minute from the *intraday* data to calculate the intraday percentage changes of stocks. The price of stock *i* at time *t* on day *d* is $p_{i,t,d}$. The log-return of stock *i* at time *t* on day *d* is $r_{i,t,d}=log\left(p_{i,t,d}\right)-log\left(p_{i,t-1,d}\right)$, as in most financial studies. This return measure is applied in the analysis on the stock-level polarity subsection.

The datasets analyzed in the current study are publicly available in the figshare.com repository and can be accessed freely at https://doi.org/10.6084/m9.figshare.5835936.v2.

## 3. Results

### 3.1. Summary Statistics of the Trading Polarity

Investigating the statistical properties of the trading polarity and its variations is crucial for understanding the underlying mechanism behind the complexity of trading behavior. This section is devoted to the summary statistics of the trading polarity.

#### 3.1.1. Market-Level Polarity

We first investigate the polarity at market-level. The polarities of the stocks are averaged to obtain the market-level polarity at a specific time. That is, the average of these polarities across the stocks in the sample serves as the polarity on the equal-weighted market portfolio to reflect the imbalance at the level of the market, that is,

(3) $${\mathrm{market}\mathrm{polarity}}_{t,d}=\frac{1}{N}\sum_{i=1}^{N}{\mathrm{polarity}}_{i,t,d},$$

where *N* is the number of stocks that are actively traded on day *d*. The summary statistics of the market polarity in the Shenzhen Stock Exchange between 4 May 2015 and 31 July 2015 on a one-minute basis are given in Panel A of Table 1.

The mean of the polarity is 0.07, suggesting that the market favor for buying man-times overwhelming the selling man-times on average. Great variations and the skewness of the distribution can be found in the standard deviation=0.11 and skewness=−0.17, which give rise to the probability of the polarity having a high variability from May to July.

When grouping the trading days into three parts as shown in Figure 3, we find that the market polarity before market crash is different in skewness. Specifically, the 0.61 skewness indicates that most of the market polarities are low, implying that buying man-times were usually very close or less than selling man-times when the market quickly went up to the peak, because the investors were trading frequently in order to benefit from the bull market. During the crash, however, the −0.11 skewness indicates that most of the market polarities are high, implying that buying man-times were usually larger than selling man-times when the investors were selling-off and selling orders were difficult to be fulfilled without multiple buying orders. One could expect that the stocks’ price movements are composed of their polarity oscillations as investors change their strategies [31]. From this oscillatory polarization in trading, a market structure will arise. We explore the related topics in the following sections.

We present the average autocorrelations of market polarity in Panel B of Table 1. As can be seen, polarity as measured by the excess number of buying man-times is highly positively autocorrelated at market-level. Therefore, there is strong evidence that a significant volume of trades in one direction is followed by further trading activity in the same direction from the macroscopic perspective. The correlation also decays fairly slowly. This evidence is consistent with previous studies [13], wherein it is pointed out that traders split their orders over time to minimize their price impact.

In addition, we find that the autocorrelations of market polarity during market crash not only are a bit higher, but also decay slower than those before crash or after crash. The differences shed lights on the distinct systematic behavior under different market conditions. While in the bear market, especially during market crash, the trading activity in one direction lasts longer than that in bull market because investors are selling-off and the market is severely lack of liquidity. The different characteristics found in different time periods may originate from the shifting of market conditions, however, may in turn reinforce the fluctuation of market status. We will explore these possibilities in the rest of this paper.

#### 3.1.2. Stock-Level Polarity

To determine whether the above findings hold for different stocks, we present the summary statistics of polarities at stock-level in Panel A of Table 2. It is shown that, though the mean of polarity are very close, the variations among stocks polarity are much larger than the market polarity across the sampling periods. Additionally, by dividing the sample period into three parts, we find that the abnormal skewness found in market-level analysis diminishes when inspecting at stock-level. The inconsistent findings between macroscopic and microscopic perspectives suggest that much attention should be paid when aggregating the trading behaviors of system components to represent the financial system behavior. Nevertheless, the positive means of polarity under different grouping strategies imply that the Chinese stock market was full of trading polarity in the observation time window.

To broaden the investigations at stock-level, we also group stocks into different subsamples according to their capitalizations. As demonstrated in Panel A of Table 2, the positive means of polarities are found across the three periods and three stock types, indicating that the probability of buying man-times more frequently overwhelmed the selling orders regardless of the capitalization of the stocks and the market conditions. In addition, we find that stocks with larger capitalization tend to have higher average polarity than others in the first and second periods. That is to say, it is easier for larger-cap stocks to have unequal numbers of man-times orders by the two trading parties than it is for the smaller-cap stocks. However, this pattern reverses after the severe market crash, where the loss of confidence has potentially transformed the trading behavior. Yet, a similar degree of variations across stocks with respect to the three stock types reveals that variations of the trading polarity are not limited to certain type of stocks in the aspect of capitalizations.

From the temporal viewpoint, the first and third periods both give evidence that larger-cap stocks have higher absolute values of left-skewness. However, in the second period, the skewness statistics are almost the same for the three types of stocks, indicating that the huge market turbulence generated systematic accordance in the distribution of trading polarity regardless of stocks’ capitalizations, and the buying polarity plays a more dominant role at the beginning of a market crash (during crash period). The transitions between the two states present results on vibrations in a collective trading pattern that originates from the changing of market anticipation.

Panel B of Table 2 shows that the average autocorrelations of stocks’ polarities is positive and decays fairly slow with the increase of time lags, though the absolute values of correlations are not as large as autocorrelations of market polarities shown in Table 1. Nonetheless, it can be similarly inferred that a significant number of trades in one direction is followed by further trading activity in the same direction from the microscopic perspective. Interestingly, the autocorrelations at stock-level during crash are overall smaller than that at the two other periods, which is just the opposite with what has been spotted at market-level. We argue that the influence of interconnections among stocks would be higher than the impacts of individual self-reinforcements when market suffers from systemic risk [3]. Yet, when aggregating the dynamics of stock polarities into market polarities, these interconnections among individuals within the system are converted to autocorrelations of dynamics of system itself. Therefore, the autocorrelations are lower at stock-level but higher at market-level during market crash.

Given these exploratory analyses, we can interpret the polarity indicator as a measure of the market trading pattern or as a measure of the market-level irrational behavior. Note that the previous studies obtain similar findings for individual stocks on a daily basis [13], here we supplement with additional evidence from high frequency data, which is of one minute basis. To deepen the understanding of the indicator, the following sections investigate not only the essence of its distinct roles but also its conjunctions with returns and investors’ emotion.

### 3.2. Polarity and Return

As polarity corporates the most basic decision units in trading stocks, we further investigate its influences on returns through time-series regressions.

#### 3.2.1. Market Polarity and Return

Here we use the SZSC index to calculate the market-level return. Specifically, we use the last price of every minute from the *intraday* data. The value of the index at time *t* on day *d* is $p_{t,d}$ and the market return at time *t* on day *d* is $MR_{t,d}=\left(p_{t,d}-p_{t-1,d}\right)/p_{t-1,d}$. In our time-series return regressions, we include the contemporaneous polarity and five lags of polarity and run the following regression for each trading day.

(4) $$MR_{t,d}=a_{d}+\sum_{k=0}^{5}b_{k,d}{\mathrm{market}\;\mathrm{polarity}}_{t-k,d}+e_{d},$$

where $a_{d}$ is interaction for day *d*’s regression, $b_{k,d}$ is the regression coefficient for polarity of lag *k* on day *d*, and $e_{d}$ is the error term. We report the average values of estimated coefficients together with the percentages of coefficients being significant over the 64 trading days in Table 3. Panel A presents results using data ranging from May 4 to July 31, whereas Panel B-D present results for the three non-overlapping periods as shown in Figure 3.

Table 3 indicates that the current polarity is negative and significant for almost half of the trading days, regardless of the market conditions as shown in Panel B-D. Recall that a high polarity means a high degree of buying man-times overwhelming selling man-times, implying that big selling orders are being fulfilled by multiple small buying orders. Thus, the contemporaneous relation between polarity and market return demonstrates the negative immediate price effects at the high-frequency level when the demands on buyer side are smaller than the supplies on seller side. While we present results for five lags of market polarity, we have checked for robustness using more lags of the above equation. The results are not significantly affected by the inclusion of more lags, especially for lagged polarities k=0,1,2,3. As can be seen in Panel A, the average coefficients on the lagged polarities k=1,2,3 are overall positive, showing that the autocorrelated polarities cause the effect of the lagged polarity to be reversed out in the current minute’s return. Though the lagged polarities k=5 have higher percent negative and significant, it should be noted that most of the coefficients for lagged polarities k≥4 are not significant when more lags included.

As the lagged polarity affect return for 3 min, the effect of autocorrelated polarities on returns plays a non-neglected role considering that the defined polarity is composed of trading imbalance in the previous one minute interval. Additionally, the lagged polarities have high proportions of positive and significant coefficients in the bull market and these proportions decay with the increase of lags, see Panel B. However, things changed since market crash, where the proportions of positive and significant coefficients become smaller and barely vary among different lags k=1,2,3. The results show that the market returns become less dependent on the very short-term previous trading activities when there are severe turbulences. On top of all these, it is revealed that the associations between market polarity and market return are different under different market conditions. We will further explore these at stock-level in the next subsection.

#### 3.2.2. Stock Polarity and Return

For a given trading polarity within one minute of one stock, the price impact could be obtained by its return within that minute. We calculate the return for each stock in every minute, and then match it with the polarity. Denote the price of stock *i* at time *t* on day *d* as $r_{i,t,d}$, where $r_{i,t,d}=log\left(p_{i,t,d}\right)-log\left(p_{i,t-1,d}\right)$, which is commonly used in most financial studies. For every $r_{i,t,d}$, there is only the polarity of $polarity_{i,t,d}$.

In the time-series return regressions, we include the contemporaneous polarity and five lags of polarity as we do in Section 3.2.1. Specifically, we run the following regression for each stock on every trading day,

(5) $$r_{i,t,d}=a_{i,d}+\sum_{k=0}^{5}b_{i,k,d}{\mathrm{polarity}}_{i,t-k,d}+e_{i,d},$$

where $a_{i,d}$ is the interaction term for stock *i*’s regression on day *d*, $b_{i,k,d}$ is the regression coefficient for lag-*k* polarity of stock *i*’s regression on day *d*, and $e_{i,d}$ is the error term. We report the average values of estimated coefficients together with the percentages of coefficients being significant for all the stocks over the 64 trading days in Table 4. Panel A presents results using data ranging from 4 May to 31 July, whereas Panel B-D present results for the three non-overlapping periods as shown in Figure 3.

The results are overall consistent with findings in the market-level analysis, with relatively lower percentages across all sample periods. As can be seen in Panel A of Table 4, the coefficients for $polarity_{i,t,d}$ are again negative, which intuitively indicate that the pattern of selling man-times overwhelming the buying man-times is more likely to cause lower current returns. The average coefficients on the lagged polarities k=1,2,3 are overall positive. This again demonstrates that the autocorrelated polarities lead to the effect of lagged polarity to be reversed out in the current minute’s return. The reversion is found in both market-level and stock-level regressions and can be concluded as one of the stable findings in the circumstances of high-frequency analysis.

Note that existing theoretical study has pointed out that this reversion arises because investors anticipate the next period’s trading imbalance due to the fact that trading in a certain direction is more likely to be followed by more trading in the same direction, which is then reflected in the premium [13]. However, the existing study only proved the theory through regressions on a daily basis. Here we not only provide evidence on a one-minute basis, but also valid the results from both market-level and stock-level. Therefore, our results imply that the theory of inventory effects in stock price movements still hold at the high-frequency facet. But it should be noted that compared with the daily examination, where the effect lasts for 5 days, here we observe a 3-minute impact. This is possibly because the polarity is refined from transactions during one minute interval and could only captures the imbalance in the corresponding small time window. From this perspective, a 3-minute impact actually plays a non-negligible role in price formation and offers informative insights for high-frequency traders in particular. Additionally, it is shown that this correlation changes daily according to the market conditions at the stock level, suggesting that the polarity could work as a reliable signal for the change of aggregate system at the macro-level even though it originates form trading decisions at the micro-level.

Interestingly, compared with the bull market (Panel B), both crash periods (Panel C and D) have lower percentages of negative and significant coefficients at lag k=0, indicating that the power of immediate price impact of the current polarity decreases. However, the percentages of positive and significant coefficients for the first-lag polarity in the two crashing periods are a bit higher than that in bull market. The difference implies that the previous one minute polarizations inspire more price impact at the stock level in market crashes than they do in the bull market. Recall that this finding does not hold at market-level (see Table 3), we argue that the inconsistency between market-level and stock-level analysis shed lights on that the trading behaviors at the micro-level could turn out to be counterintuitive at the macro-level in the corresponding aggregate system. Nevertheless, these results further indicate that the rapid response of the market, particularly in different levels, can be comprehensively captured by the well-resolved “polarity” indicator in one-minute granularity under the high-frequency background.

### 3.3. The Flipping of Polarity and Stock Returns

From the previous analysis, one could expect that the polarity constantly switch their signs; they could be positive, negative, or zero. For instance, once it has been flipped, a former selling polarity becomes a subsequent buying polarity in a subsequent downtrend. Similarly, once a balanced polarity has been penetrated, it becomes either a selling polarity or a buying polarity in a later phase. In this section, we propose two new indicators to capture the dynamics of polarity flipping to depict the transitions of trading behavior at the very micro-level in stock market.

We first investigate the effect of the polarity flipping amplitudes, which is called the flipping depth. Define the flipping depth of stock *i* on day *d* as

(6) $${\mathrm{flipping}\;\mathrm{depth}}_{i,d}=\sum_{t}|{\mathrm{polarity}}_{i,t,d}-{\mathrm{polarity}}_{i,t-1,d}|,$$

where *t* is the time at which the polarity flips on day *d*. This depth reveals the strength of the investors’ trading desire shifting.

In addition, the time required for the trading polarity to break out of the dominant direction of the polarity is of great interest. We call this time the *length before flipping*. For example, suppose we have a five-minute polarity series such as (0.2,−0.3,−0.4,−0.2,0.3). Then, the negative polarity length is three, as the number of negative polarities between two positive polarities is three. The length before flipping reflects how long the negative polarity consecutively has domination. Specifically, a negative length of stock *i* encompasses two flips in the time span (as we have the polarities equidistantly recorded at a one-minute frequency) with a negative polarity in between. The same applies to the positive polarity length.

We now focus on the influence of stock return arising from the flipping of polarity by involving the above two indicators in the following regression for each trading day as

(7) $$\begin{array}{cc} r_{i,d}-MR_{d}= & a_{d}+b_{1d}{\mathrm{depth}}_{i,d}+b_{2d}{\mathrm{positive}\;\mathrm{length}}_{i,d}\\ & +b_{3d}{\mathrm{negative}\;\mathrm{length}}_{i,d}+b_{4d}{\mathrm{polarity}}_{i,d}+e_{d}, \end{array}$$

where the dependent variable $r_{i,d}-MR_{d}$ is the daily excess return of stock *i*, ${\mathrm{polarity}}_{i,d}$ is the daily averaged polarity that obtained from the one-minute polarity time series of stock *i*, ${\mathrm{depth}}_{i,d}$ is the daily cumulative flipping depth of polarity, $a_{d}$ is the interraction, $b_{id},i=1,2,3,4$ are regression coefficients and $e_{d}$ is the error term. Length before flipping indicators are denoted as ${\mathrm{positive}\;\mathrm{length}}_{i,d}$ and ${\mathrm{negative}\;\mathrm{length}}_{i,d}$, respectively. As our focus is on the difference of polarity related indicators’ effects under different market conditions, the regression in Equation (6) is therefore designed to be conducted for each trading day *d*, which makes it a cross-sectional analysis instead of time series analysis for stocks. Besides, with all the stocks listed on Shenzhen Stock Exchange considered in regressions, the sample size is greater than one thousand, and thus the normality assumption becomes less important as linear regression models are fairly robust to violations of the normality assumption in large sample size settings [32]. However, considering the potential effects of violations to regression assumptions, we use Generalized Least Squares (GLS) to estimate regression coefficients. GLS is known as a general solution when there is a certain degree of correlation between the residuals in a regression model [33]. The results are summarized in Table 5.

As can be seen, the price impact of polarity is negative, implying that a higher contemporaneous imbalance of selling and buying orders will lead to lower stock returns on a daily basis. This is in line with the regression results at the minute-level in Section 3.2.2. While economic variables are known to move asymmetrically over the business cycle: quickly and sharply during crises, but slowly and gradually during recoveries [34], the signs and magnitudes of ${\mathrm{polarity}}_{i,d}$ are very close across the three different market conditions, implying it has coherent effect on stocks’ daily excess returns. The flipping depth is also negatively associated with stock returns with large proportions of coefficients being significant. Note that the flipping depth reflects the degree to which the polarity shifting, its negative relation with returns indicates that the swing of disagreement of trading direction among investors draws the price down in most cases. Additionally, the average coefficients of ${\mathrm{flipping}\;\mathrm{depth}}_{i,d}$ switch signs after market crash. However, the asymmetry effect is subtle because the ratios of significant coefficients also decline.

As for the length before flipping, it is shown that higher negative lengths will lead to lower returns. That is to say, the longer the selling polarity dominants the buying polarity, the lower the daily return of the stock will be. The positive flipping lengths are just the opposite. We will show in Section 3.4 that the vibration of positive flipping lengths that represents the subtle changing of trading directions at the micro-level indeed offers few implications and in particular, is not necessarily associated with the transition of market conditions. It is also worth noticing that the proportions for coefficients of these explanatory variables to be significant are lower in the “post-crash” period than those in the other two. On one hand, this implies that we can hardly regard the sign reversing of ${\mathrm{negative}\;\mathrm{length}}_{i,d}$ as asymmetry effect, similar to that of ${\mathrm{flipping}\;\mathrm{depth}}_{i,d}$. On the other hand, it again reminds us the difficulty of interpreting and predicting the market behaviors after crash.

### 3.4. Using Polarity to Signal the Market Changes

The previous results demonstrate the power of polarity indicator in enabling us to recognize changes of trading behaviors. The anomaly of these changes will further provide insights into the unusual behavior of a market. In this section, we explore how to use the proposed indicator to signal market crash.

#### 3.4.1. The Changing of the Polarity-Return Correlation

**Figure 4:**
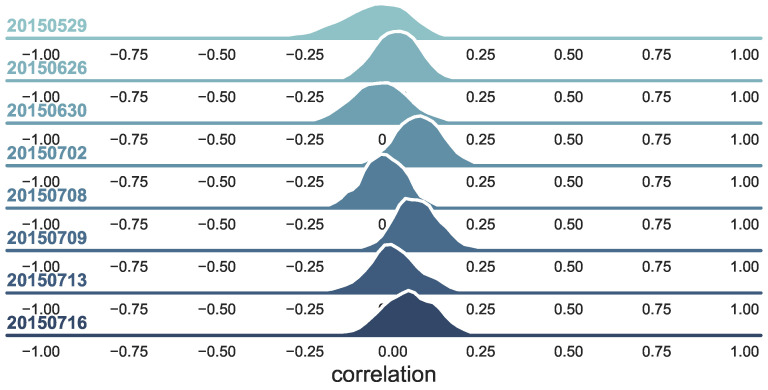
The correlation distribution over a couple of days. Using the one-minute frequency data, we obtain the whole set of polarity-return correlation coefficients for all the stocks on day *d*. These trading days are representatives for different market conditions. May 29 belongs to the bull market period (before crash). On 26, 30 June, and 2 July, over one thousand stocks hit the price limit of −10%, and these trading days belong to the crash period (during crash). The rest belong to the after crash period.

In Section 3.2.2, we find that the percentage of coefficients being negative and significant for contemporaneous polarity varied across different periods, which inspires us using the movements of their relationships to signal market instability. By constructing the Pearson correlation coefficient of the polarities and log-returns of each stock on each day using the one-minute frequency data, we obtain the whole set of correlation measures for all the stocks on day *d*, and this set’s probability distribution is denoted as $Q_{d}\left(x\right)$. Figure 4 shows the correlation distributions of a few days that are on behalf of different market conditions, which basically demonstrates that the correlations vary from time to time; some of them have left skewness, but others have right skewness; whereas some are fat-tailed, others are not. As the trading polarity originates from the market microstructure, we argue that the microstructure would have changed under various market conditions.

To assess the disparity of the correlation distribution from day to day, we construct Kullback–Leibler divergence measures on the daily correlation coefficients’ distribution. We denote the probability distributions on days *d* and d−1 as $Q_{d}\left(x\right)$ and $Q_{d-1}\left(x\right)$. The Kullback–Leibler divergence quantities the similarity of two distributions by the difference between cross entropy of the two, that is, $-\sum_{x}Q_{d}\left(x\right)\log\left(Q_{d-1}\left(x\right)\right)$, and the entropy of the benchmark distribution $Q_{d-1}\left(x\right)$, that is, $-\sum_{x}Q_{d}\left(x\right)\log Q_{d}\left(x\right)$. In other words, the Kullback–Leibler divergence is measured to be from $Q_{d-1}$ to $Q_{d}$ and is defined by

(8) $$KL(Q_{d}\|Q_{d-1})=\sum_{x}Q_{d}\left(x\right)\log\frac{Q_{d}\left(x\right)}{Q_{d-1}\left(x\right)}.$$

**Figure 5:**
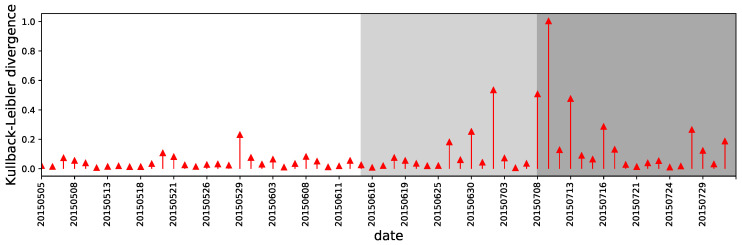
The Kullback–Leibler(KL) divergence from $Q_{d-1}\left(x\right)$ to $Q_{d}\left(x\right)$. The higher the KL divergence is, the more diverse the correlation distribution will be compared to the prior day. It is clear that the KL divergence increases in crash and post-crash periods.

The divergence of the correlation distributions is shown in Figure 5. The shaded areas correspond to the stock market crisis from June 2015 to July 2015. The figure clearly shows that the KL divergence increases in difficult times, which widens the difference from the prior day. The first notable difference is observed on 26 June, when the market suffered from over one thousand stocks falling to their lower limits. The most significant changes occurred around the beginning of the post-crash times, which is when the government began to take measures to save the market. From this point of view, the measure is efficient in indicating the phase transitions in the underlying correlation between trading behavior and stock returns. Thus, the measure could be used to signal the changing of the market.

#### 3.4.2. How Does the Flipping Polarity Relates to Market Changes?

The trading polarity oscillations or vibrations will exhibit a rhythm, which potentially configures the different market patterns produced by the interplay between buyers and sellers. We have discuss this by defining the depth and length of polarity flipping in Section 3.3. We now explore how the flipping polarity varies in depth and length from micro-oscillations to a bull or bear market.

- Flipping Depth

**Figure 6:**
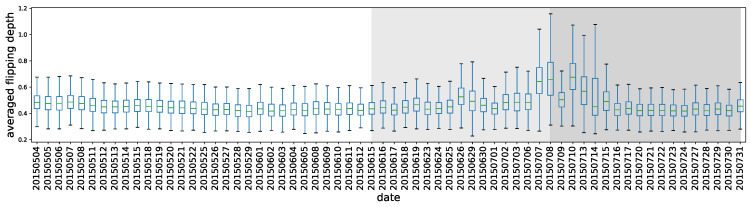
Polarity flipping depth. The box plot graphically depicts groups of the numerical depths of 1646 stocks on the corresponding day through their five-number summaries: the smallest observation, lower quartile (Q1, 25th percentile), median (Q2, 50th percentile), upper quartile (Q3, 75th percentile), and largest observation. If we denote the spread between Q3 and Q1 as *h*, then the outliers are defined as those less than Q1−1.5h or greater than Q3+1.5h. In each box with this representation, outliers are ignored to make the graph clear.

For comparison reasons, we divide the daily cumulative flipping depth by the flipping times and obtain the *averaged flipping depths*, which are caused by cumulative polarity flipping from either the selling or the buying party to the counterparty. Figure 6 gives the whole picture of the day-to-day averaged flipping depth distribution. Each box represents the depths of 1646 stocks on the corresponding day. It is obvious that the pre-crash and post-crash periods demonstrate significantly unusual behavior with the corresponding boxes rising. The interesting change of the phase behaviors of the market system could be viewed as the outcome of transitions in both investors’ expectations and their trading preferences, as observed in a complex adaptive system [35]. Observe that the first significant change occurred on 26 June 2015, in which the stock market had an abrupt and sharp fall. It is also apparent that at the beginning of the post-crash period, the markets functioned differently. The amplitude of the investors’ trading polarity shifting became larger, and the variation of this amplitude itself grew, indicating that there were severe imbalances in the selling and buying activities. The extent of the imbalance varied among the stocks.

- Length before Flipping

**Figure 7:**
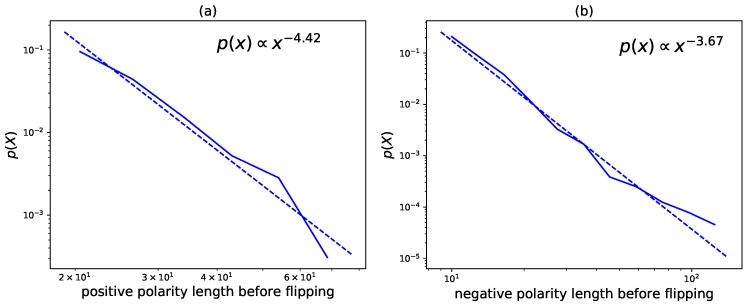
Probability density function (p(X), blue) of the length before flipping and the fitted power-law distribution on 8 May 2015. Subfigure (**a**) shows the positive flipping length distribution of all the stocks, where the positive flipping length is defined as the time span between two positive polarities. Subfigure (**b**) shows the negative flipping length distribution of all the stocks, where the negative flipping length is defined as the time span between two negative polarities. The dashed lines are power-law fittings.

**Figure 8:**
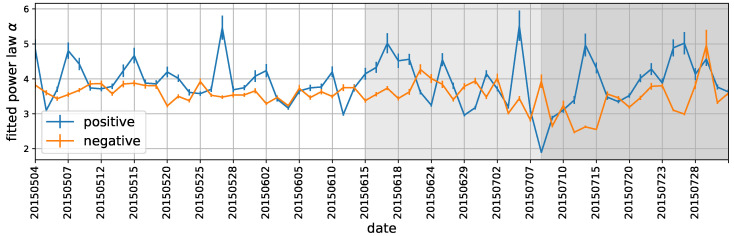
The fitted power-law exponent α of the positive and negative flipping length distribution for each day. The daily distribution is a mixture of all the stocks’ daily flipping lengths. The error bars are the estimated standard errors for α on the corresponding day.

When we mix all the stocks’ daily flipping lengths together, we obtain the distribution of the flipping lengths for each day. Figure 7 is an example. Surprisingly, we find that the distribution is a well fitted power-law (the parameter estimation procedure follows [36]) for both positive flipping lengths and negative flipping lengths. As we observe similar effects not only on 8 May 2015, but also on other days in our dataset, we conclude that the power-law scaling behavior of the polarity flipping length is a universal feature. We plot the trend of the daily power-law exponent in Figure 8. As can be seen, whereas the negative trend changes from the pre-crisis period to the crisis period, the positive trend has vibrations in all three periods. This finding shows that the two trends play completely different roles. Nevertheless, most of the fitting exponents are between 3 and 5, implying a prevalent law of heavy tails in the flipping length.

Note that the flipping length in essence can be treated as the time interval between two consecutive flips either from negative to positive polarity or vice versa. Hence, the power-law distribution implies that the domination direction of the polarity is bursty. Bursts, which have been observed in a wide range of human-related systems, indicate enhanced activity levels over short periods of time followed by long periods of inactivity [37]. In the present scenario, the observed burstiness indicates rapid vibrations between the two directions of polarity separated by long periods of one direction’s domination. Following Goh and Barabási [38], we use the burstiness parameter $B=\frac{\sigma_{\tau }-\mu_{\tau }}{\sigma_{\tau }+\mu_{\tau }}$ to obtain the extent of the burstiness for the flipping lengths, where $\mu_{\tau }$ and $\sigma_{\tau }$ are the mean and standard deviation of the daily power-law distributions, respectively. The magnitude of the parameter correlates with the signal’s burstiness. When B=1, the series is the most bursty signal possible in this scenario. When B=0, the series is neutral, and B=−1 corresponds to a completely regular (periodic) signal.

**Figure 9:**
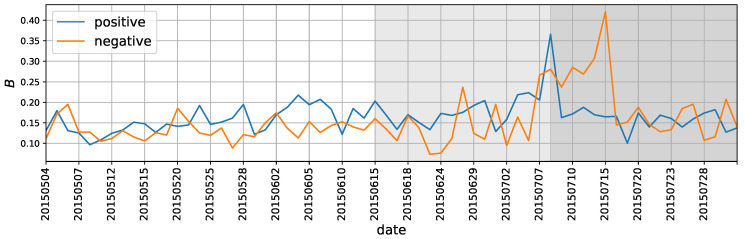
The burstiness parameter *B* for the daily distributions of positive and negative flipping lengths. The daily distribution is a mixture of all the stocks’ daily flipping lengths.

The trends of burstiness can be found in Figure 9. In the pre-crash period, both the positive and negative flipping length distributions have roughly stable performance. A negative flipping length occurred in the first burstiness hop on 26 June, when the market experienced the most severe crash. The higher value of the burstiness revealed by the hop implies that the selling polarity of the stocks prevailed longer in the trading hours, which is consistent with the enduring panic selling that happened on that day. Furthermore, at the beginning of the post-crash period, the burstiness of the negative flipping length became larger than before, indicating that the market sell-off was growing more severe.

Interestingly, the burstiness of the positive and negative flipping lengths reached a high level at the same time on 8 July, when China’s state-owned Assets Supervision and Administration Commission prohibited state-owned companies from reducing their stock-holding shares and the China Securities Regulatory Commission declared an increase in the purchasing of mid-cap and small-cap stocks. These decisions conveyed the notion that the powerful bailout measures announced on that day had led the trading behavior to be poles apart among stocks based on whether the selling man-times dominated the trading or the buying man-times dominated the trading. This divergent behavior was probably due to the co-existence of two polarized expectations in the face of government bailout: whereas some people were encouraged by the “national team” and decided to buy, others decided to take the opportunity to sell their holdings. However, the burstiness of the positive flipping length dropping sharply on the following day demonstrates that the market’s confidence did not last longer than expected.

It seems that the burstiness parameters are more sensitive than the power-law exponents in the observed system, as significant differences are exhibited in the pre-crash, crash, and post-crash periods. Furthermore, the observed bursty character reflects some fundamental and potentially generic feature of the market participants’ trading dynamics during market interventions. This feature again demonstrates that it is feasible to develop a signal that could systematically reflect the trading psychology and emotion through the proposed trading polarity, which originates from micro-level data.

- Using entropy to characterizing the state of market

We have discussed how the flipping polarity relates to market changes through either fitting the distributions or presenting box plots of *flipping depth* and *length before flipping*. Here we further develop the entropy measures on a daily basis for these two indicators to aggregate stocks’ trading imbalance at macro level. As they are continuous variables by definition, the differential entropy for a random variable is used, that is,

(9) H(X)=−∫f(x)logf(x)dx,

where *x* denotes either *flipping depth* or *length before flipping*, f(x) is the probability density function for each trading day. A simplest estimator for (9) is

(10) $$H\left(X\right)\approx \frac{1}{N-1}\sum_{i=1}^{N-1}\log\left(x_{i+1}-x_{i}\right)+\phi \left(1\right)-\phi \left(N\right),$$

where ϕ(x) is the digamma function, $\phi \left(x\right)=\Gamma {\left(x\right)}^{-1}d\Gamma \left(x\right)/dx$. It satisfies the recursion $\phi \left(x+1\right)=\phi \left(x\right)+\frac{1}{x}$ and ϕ(1)=−C, where *C* is the Euler-Mascheroni constant [39].

**Figure 10:**
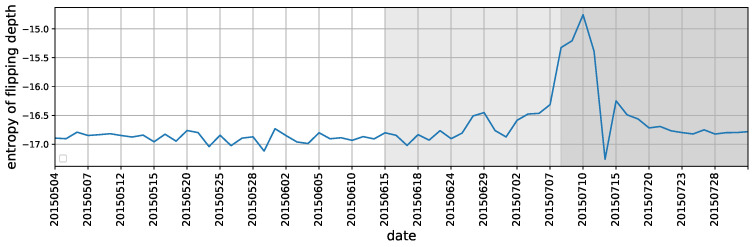
The differential entropy for the daily distributions of flipping depth.

**Figure 11:**
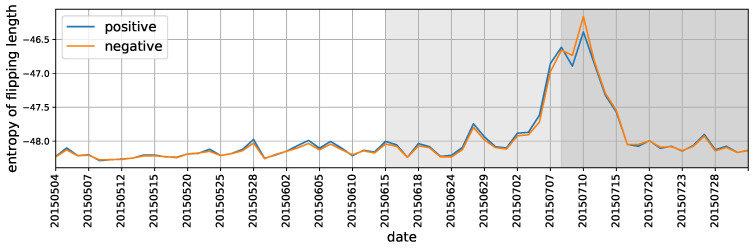
The differential entropy for the daily distributions of length before flipping.

The results of entropy for flipping depth and flipping length under Equation (10) are shown in Figure 10 and Figure 11. As can be seen, the entropy measures successfully capture the market transitions from the period of “during crash” to the period of “after crash”. Another changing point is around June 26, 2015, when the stock market had an abrupt and sharp fall. These results are also consistent with our previous measures based on fitting distribution parameters and visualization (see Figure 5 and Figure 6 and Figure 8 and Figure 9), implying that entropy of polarity flipping can also signal the market systematical variations, which in turn facilitates the usefulness of proposed polarity and its extensive indicators in reflecting some fundamental and potentially generic feature of the market participants’ trading dynamics.

Overall, the discussions on the polarity flipping behavior have shed light on the trading polarities’ essential nature in micro trading behavior. In the next section, we consider its interconnections with investors’ emotion and stocks’ returns from the perspectives of the market level as well as the stock level.

#### 3.4.3. Market Polarity and Emotions

The above analysis is based on data within the financial system, including stock prices, the trend of market index and transaction records. We now step further to investigate the polarity’s interconnection with social media emotion that is refined from outside the financial system.

**Figure 12:**
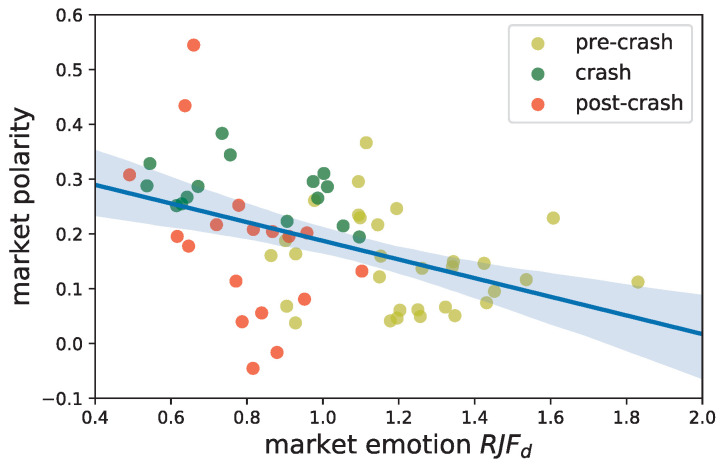
The trading polarity value correlated with the market emotion indicator. Based on the online emotions of investors, $RJF_{d}$ on the *x*-axis is defined as the ratio of joy (greed) to fear (RJF) on day *d*, $RJF_{d}=\frac{X_{joy,d}}{X_{fear,d}}$. The market polarity on the *y*-axis is the average polarity of the stocks when the SZSC index reaches its daily minimum.

The daily market emotion of the Chinese stock market is measured by $RJF_{d}$ [1]. While $RJF_{d}$ is greater than 1, the investors are optimistic and consider the market to be rising. However, when $RJF_{d}<1$, investors are irrational, fear is the dominant emotion in the market, and investors are afraid of the loss of benefit. The investors stay rational and the emotions are stable if $RJF_{d}$ is approximately 1. The daily $RJF_{d}$ is shown on the *x*-axis in Figure 12.

Here we use the market polarity on a daily basis as introduced in Section 3.1.1. We consider the SZSC index to represent the market trend. From the *intraday* one-minute index return series, we select the minimum return on each day, and we use the market polarity at that same moment to represent the daily polarity. The reason is that as Zhou et al. [1] has noted, the online emotion is more sensitive to a poor market state. Therefore, the polarity when the market reaches the lowest point could serve as the daily polarity to adjust to the daily market emotion. The polarities are exhibited on the *y*-axis in Figure 12.

Figure 12 presents a scatter plot between the emotion and the polarity, along with the line of best fit. We find that most of the market polarity is above zero, which implies that the buying polarity is the dominant role when the market reaches its worst point of each day. More importantly, the figure illustrates the negative correlation between the emotion indicator and the market polarity. That is, as the market becomes less optimistic, the polarity moves away from the balanced states to a buying polarity, which suggests that a generic buying opinion is embedded into the market.

The yellow dots are those in the pre-crisis period when the market continued climbing, and the market emotion is excited most of the time. The green and red dots are from the crash and post-crash periods, respectively, and they depart from the fitted line. If we separately compute the correlation coefficients for the three period, we obtain -0.197, -0.382, and -0.493. Although the number of data points in every period may not be sufficient to obtain a linear fit, we could still roughly find that the correlation pattern grows sharper in the crisis period and in the post-crisis period.

It may be claimed that accumulating enough expressions, such as texts in social media [1,40] or queries in search engines [41], is always necessary for the direct measurement of the market sentiment. However, in the circumstance of high-frequency analysis, collecting these data for a sentiment analysis is unfortunately challenging due to the severe sparsity of emotional expressions in such a short interval. The significant correlation disclosed here implies that our presented polarity, which is essentially calculated in the context of high-frequency trading, can break the above constraints and reflect the sentiment of the market in real time.

## 4. Conclusions and Discussion

With the rapid growth of financial data and the magnificent developments in computational ability, studies driven by big data and dense computing in recent decades offer better understandings of the financial market [16,19,25]. Rather than the heavy dependence on theoretical assumptions, data-driven solutions can model the market more systematically and realistically, enabling real-time and precise reflections or even predictions of financial systems in real-world scenarios [14,15,27].

Under these circumstances, we propose an indicator that we call the trading polarity to depict the imbalanced relationship between buying and selling in the granularity of man-times. From the initial investigations, we find that it is positively autocorrelated at both market-level and stock-level. With regard to the relationship between polarities and returns, our regression analysis mainly follows a previous study and obtain similar conclusions at minute granularity in addition to their daily examinations Chordia and Subrahmanyam [13]. The results show that the autocorrelated polarities cause a positive relation between lagged polarities and returns, while the current polarity has the opposite effect. This discovery sheds light on the role of inventory effect in stock price movements on a minute basis and contributes to the understanding of trading imbalance from the very high-frequency perspective. We also perform analysis that goes beyond a tranquil market and provide more implications of the specific relation before, during and after market crash.

Furthermore, we have assemblies of indicators that reveal insight into the trading polarity, including the polarity flipping depth, flipping length, and distribution of correlations between a stock’s polarity and return. We have shown that these measures could bear a more meaningful relation to changing market conditions, especially during an extreme market crash. The usefulness of this framework is that it deepens our understanding of trading patterns, which originates from “micro” data but surprisingly possesses explanatory power at the “macro” level. Moreover, discovering the vibratory rate of a market gives one the key to trading within it efficiently. More importantly, we find a convincing correlation between the market polarity and the market emotion. This correlation again implies that the proposed trading polarity, which can easily be calculated in the context of high-frequency data, can provide a measurement of the sentiment for the market in real time. Thus, this correlation could serve as an effective behavioral signal at the market level.

The proposed polarity could be of interest to market regulators and investors as it is an indicator that works for tracking both micro and macro trading patterns. Broadly speaking, regulators should be aware of the polarity in the stock market. First, attempts are needed to regulate those imbalanced polarities that are likely to push the price away from fundamental prices or potentially increase the crash risk. Second, regulators should gain knowledge of the polarity patterns in stock trading both as a whole and in cases where there are externalities from traders to market prices and real economic activity. These issues call for more research with longer-term data in the future. Furthermore, the appropriate method to integrate the volume into the current analysis is also important given that the trading activity has usually been proxied by the volume [28], and further research should consider this issue in the presented framework. In addition, if data are available, we could compare the different characteristics of markets in different countries. It is also possible that the trading polarity could be associated closely with the financial risk of both individual stock and entire market and would be specifically explored in the future work.

## Figures and Tables

**Table 1:** Summary statistics of ${\mathrm{market}\;\mathrm{polarity}}_{t,d}$.

**Panel A: Descriptive Statistics**								
	**Mean**			**Std.dev.**			**Skewness**	
4 May 2015–31 Jul 2015	0.07			0.11			− 0.17	
pre-crash	0.07			0.06			0.61	
crash	0.14			0.10			− 0.11	
post-crash	0.02			0.13			− 0.14	
**Panel B: 1 min Autocorrelations**								
	**lag 1**	**lag 2**	**lag 3**	**lag 4**	**lag 5**	**lag 10**	**lag 15**	**lag 30**
4 May 2015–31 Jul 2015	0.92	0.87	0.82	0.77	0.72	0.55	0.45	0.21
pre-crash	0.92	0.86	0.80	0.74	0.69	0.49	0.39	0.12
crash	0.93	0.89	0.84	0.80	0.77	0.64	0.56	0.34
post-crash	0.93	0.88	0.82	0.78	0.75	0.60	0.48	0.26

Note: In Panel A, the summary statistics represent the averages of all the one-minute market polarities in the Shenzhen Stock Exchange over the specified periods. In Panel B, the autocorrelaitons are the averages of the correlations for one-minute time-series market polarity in each day, that is, $autocorrelation_{lag,d}=cor\left({\mathrm{market}\;\mathrm{polarity}}_{t-lag,d},{\mathrm{market}\;\mathrm{polarity}}_{t,d}\right)$ and $autocorrelation_{lag}=\frac{1}{D}\sum_{d=1}^{D}autocorrelation_{lag,d}$, where *D* is the number of trading days. Here we investigate the autocorrelations with lags of 1–5, 10, 15, and 30 min, separately.

**Table 2:** Summary statistics of stock ${\mathrm{polarity}}_{i,t,d}$.

**Panel A: Descriptive Statistics**								
		Mean	Std.dev	Skewness	Cap	Mean	Std.dev	Skewness
4 May–31 Jul		0.08	0.34	−0.17	small	0.07	0.33	−0.13
					mid	0.08	0.34	−0.18
					large	0.08	0.34	−0.25
pre-crash		0.07	0.33	−0.22	small	0.06	0.33	−0.16
					mid	0.07	0.33	−0.23
					large	0.08	0.32	−0.31
crash		0.14	0.34	−0.11	small	0.12	0.34	−0.11
					mid	0.14	0.34	−0.11
					large	0.14	0.34	−0.11
post-crash		0.04	0.35	−0.18	small	0.05	0.34	−0.14
					mid	0.04	0.35	−0.18
					large	0.02	0.36	−0.25
**Panel B: 1 min autocorrelations**								
	lag 1	lag 2	lag 3	lag 4	lag 5	lag 10	lag 15	lag 30
May–Jul	0.46	0.31	0.25	0.21	0.20	0.15	0.10	0.03
pre-crash	0.46	0.31	0.25	0.21	0.19	0.15	0.09	0.03
crash	0.47	0.29	0.22	0.20	0.20	0.12	0.10	0.01
post-crash	0.45	0.32	0.27	0.22	0.21	0.15	0.11	0.04

Note: In Panel A, the summary statistics represent the averages of all the one-minute polarities for all stocks listed in the Shenzhen Stock Exchange over the specified periods. In Panel B, the autocorrelations are the cross-sectional averages of the correlations for one-minute time-series stock polarity. For stock *i* on day *d*, $autocorrelation_{i,lag,d}=cor\left({\mathrm{polarity}}_{i,t-lag,d},{\mathrm{polarity}}_{i,t,d}\right)$, and for stock *i*, $autocorrelation_{i,lag}=\frac{1}{D}\sum_{d=1}^{D}=autocorrelation_{i,lag,d}$. Then we can calculate the mean of autocorrelations by averaging over stocks, that is, $autocorrelation_{lag}=\frac{1}{N}\sum_{i=1}^{N}=autocorrelation_{i,lag}$, where *D* is the number of trading days, *N* is the number of stocks. Here we investigate the autocorrelations with lags of 1-5, 10, 15, and 30 min, separately. Also, results of autocorrelations for different types of stock in the aspect of capitalizations are very similar to those in Panel B. These results are available upon request.

**Table 3:** Regressions of intraday one minute market returns $MR_{t,d}$ on ${\mathrm{market}\mathrm{polarity}}_{t-k,d}$.

**Panel A: 4 May to 31 Jul.**				
lag *k*	average coefficient	percent positive	percent positive and significant	percent negative and significant
0	−0.0061	14.06%	0.00%	43.75%
1	0.0025	73.44%	23.44%	1.56%
2	0.0017	67.19%	21.88%	6.25%
3	0.0018	67.19%	12.50%	1.56%
4	0.0009	65.63%	7.81%	4.69%
5	−0.0007	45.31%	3.13%	12.50%
**Panel B: 4 May to 14 Jun (pre-crash).**				
lag *k*	average coefficient	percent positive	percent positive and significant	percent negative and significant
0	−0.0068	13.33%	0.00%	53.33%
1	0.0034	86.67%	40.00%	0.00%
2	0.0031	73.33%	30.00%	0.00%
3	0.0019	73.33%	10.00%	0.00%
4	0.0005	53.33%	3.33%	3.33%
5	−0.0012	46.67%	3.33%	16.67%
**Panel C: 15 Jun to 7 Jul (crash).**				
lag *k*	average coefficient	percent positive	percent positive and significant	percent negative and significant
0	−0.0084	13.33%	0.00%	33.33%
1	0.0039	80.00%	13.33%	6.67%
2	0.0013	73.33%	13.33%	6.67%
3	0.0018	53.33%	13.33%	0.00%
4	0.0019	73.33%	6.67%	6.67%
5	−0.0014	33.33%	0.00%	20.00%
**Panel D: 8 Jul to 31 Jul (post-crash).**				
lag *k*	average coefficient	percent positive	percent positive and significant	percent negative and significant
0	−0.0029	16.67%	0.00%	38.89%
1	−0.0002	44.44%	5.56%	0.00%
2	−0.0006	50.00%	11.11%	16.67%
3	0.0017	72.22%	16.67%	5.56%
4	0.0011	83.33%	16.67%	5.56%
5	0.0008	55.56%	5.56%	0.00%

Note: The two market-level indicators are on a one-minute basis. We have checked for robustness using more lags of the above equation and the results are not significantly affected by the inclusion of more lags. In particular, the coefficient results for lagged polarities k=0,1,2,3 are independent to the settings.

**Table 4:** Regressions of intraday one minute returns $r_{i,t,d}$ on $polarity_{i,t-k,d}$.

**Panel A: 4 May to 31 Jul**				
lag *k*	average coefficient	percent positive	percent positive and significant	percent negative and significant
0	−0.0009	23.13%	1.55%	31.45%
1	0.0008	77.22%	16.27%	0.54%
2	0.0006	75.40%	13.70%	0.54%
3	0.0003	64.62%	7.70%	1.33%
4	0.0000	54.08%	3.69%	2.31%
5	0.0000	50.36%	2.69%	2.76%
**Panel B: 4 May to 14 Jun (pre-crash).**				
lag *k*	average coefficient	percent positive	percent positive and significant	percent negative and significant
0	−0.0013	10.92%	0.32%	46.13%
1	0.0004	70.16%	11.19%	0.87%
2	0.0006	80.01%	16.83%	0.34%
3	0.0004	74.34%	11.43%	0.49%
4	0.0002	62.16%	5.34%	1.17%
5	0.0001	54.57%	3.33%	2.01%
**Panel C: 15 Jun to 7 Jul (crash).**				
lag *k*	average coefficient	percent positive	percent positive and significant	percent negative and significant
0	−0.0007	34.23%	2.43%	18.71%
1	0.0012	82.60%	18.34%	0.23%
2	0.0007	72.24%	11.44%	0.58%
3	0.0002	57.89%	4.59%	1.66%
4	−0.0001	48.54%	2.19%	2.88%
5	−0.0001	45.72%	1.98%	3.57%
**Panel D: 8 Jul to 31 Jul (post-crash).**				
lag *k*	average coefficient	percent positive	percent positive and significant	percent negative and significant
0	−0.0005	38.02%	3.31%	13.46%
1	0.0013	87.20%	25.33%	0.13%
2	0.0005	68.92%	9.50%	0.94%
3	0.0000	50.53%	2.91%	2.80%
4	−0.0002	42.34%	1.65%	4.19%
5	−0.0001	45.71%	2.02%	3.63%

Note: We use the one-minute frequency for the two stock-level indicators in regression analysis. While we present results for all stocks, we have also checked for robustness by dividing stocks into groups according to capitalization. The results are not significantly affected by this division.

**Table 5:** Regressions of variables related to the flipping of polarity on stock excess returns.

	Variable	Average Coefficient	Percent Positive	Percent Positive and Significant	Percent Negative and Significant
May–Jul	positive length	0.0010	81.0%	49.2%	12.7%
	negative length	−0.0021	17.5%	11.1%	49.2%
	depth	−0.0003	19.0%	3.2%	65.1%
	polarity	−0.202	0%	0%	100%
pre-crash	positive length	0.0008	73.3%	43.3%	13.3%
	negative length	−0.0029	6.7%	0.0%	53.3%
	depth	−0.0004	10%	3.3%	70%
	polarity	−0.2221	0%	0%	100%
crash	positive length	0.0010	93.3%	66.7%	6.7%
	negative length	−0.0031	26.7%	20%	60%
	depth	−0.0006	13.3%	0%	80%
	polarity	−0.2118	0%	0%	100%
post-crash	positive length	0.0015	83.3%	44.4%	16.7%
	negative length	0.0001	27.8%	22.2%	33.3%
	depth	0.0000	38.9%	5.6%	44.4%
	polarity	−0.1602	0%	0%	100%

Notes: We have removed observations with extreme values of $r_{i,d}-MR_{d}$, that is, $∥r_{i,d}-MR_{d}∥>8.5\%$ as the price limit in China stock market is 10%, to eliminate the side effect of outliers for regression analysis.
